# cIAP1/2 Are Direct E3 Ligases Conjugating Diverse Types of Ubiquitin Chains to Receptor Interacting Proteins Kinases 1 to 4 (RIP1–4)

**DOI:** 10.1371/journal.pone.0022356

**Published:** 2011-09-12

**Authors:** Mathieu J. M. Bertrand, Saskia Lippens, An Staes, Barbara Gilbert, Ria Roelandt, Jelle De Medts, Kris Gevaert, Wim Declercq, Peter Vandenabeele

**Affiliations:** 1 Department for Molecular Biomedical Research, VIB, Ghent, Belgium; 2 Department of Biomedical Molecular Biology, Ghent University, Ghent, Belgium; 3 Department of Medical Protein Research, VIB, Ghent, Belgium; 4 Department of Biochemistry, Ghent University, Ghent, Belgium; University of Illinois at Chicago, United States of America

## Abstract

The RIP kinases have emerged as essential mediators of cellular stress that integrate both extracellular stimuli emanating from various cell-surface receptors and signals coming from intracellular pattern recognition receptors. The molecular mechanisms regulating the ability of the RIP proteins to transduce the stress signals remain poorly understood, but seem to rely only partially on their kinase activities. Recent studies on RIP1 and RIP2 have highlighted the importance of ubiquitination as a key process regulating their capacity to activate downstream signaling pathways. In this study, we found that XIAP, cIAP1 and cIAP2 not only directly bind to RIP1 and RIP2 but also to RIP3 and RIP4. We show that cIAP1 and cIAP2 are direct E3 ubiquitin ligases for all four RIP proteins and that cIAP1 is capable of conjugating the RIPs with diverse types of ubiquitin chains, including linear chains. Consistently, we show that repressing cIAP1/2 levels affects the activation of NF-κB that is dependent on RIP1, -2, -3 and -4. Finally, we identified Lys51 and Lys145 of RIP4 as two critical residues for cIAP1-mediated ubiquitination and NF-κB activation.

## Introduction

Cells are continuously confronted with stress signals that initiate at the cell surface or within the cell. The ability to transduce the signal and to activate a cellular response, either through transcription-dependent or –independent mechanisms, relies on the recruitment of adaptor proteins to the extra- and intra-cellular sensors. Among these adaptors, members of the receptor-interacting protein kinase (RIP) family have emerged as essential mediators of cellular stress. All RIP kinases contain a kinase domain that is conserved between the family members and bear unique domains that allow protein-protein interactions[1]. RIP1, the first identified member of the RIP kinase family, was shown to participate in the signaling pathways activated downstream of several members of the TNF receptor superfamily[2], Toll-like receptors (TLR) 3 and 4[2], [3], and after genotoxic stress[4]. RIP1 can mediate gene transcription through activation of the MAPK and NF-κB signaling pathways, and can induce cell death by the formation of death complexes. Similarly, RIP2 (CARDIAK/RICK) was shown to participate in the activation of the MAPK and NF-κB signaling pathways downstream of the innate immune pattern recognition receptors NOD1 and NOD2[5], [6], [7], and to be part of a pro-apoptotic complex activated by NOD1[8]. RIP2 is also required for optimal T-Cell Receptor (TCR) signaling and, although controversial, was reported to mediate TLR responses[5], [6], [7], [9]. Mice lacking RIP3 do not develop spontaneous phenotypic manifestations[10], but this kinase has been reported to be a major player in the execution of a caspase-independent type of cell death called necroptosis[11], [12], [13]. In addition, the ectopic expression of RIP3 in cells was shown to induce apoptosis and NF-κB activation[14], [15]. RIP4 (PKK/DIK) also induces JNK and NF-κB when ectopically expressed[16], [17], but the pathways that lead to activation of RIP4 remain unidentified. Transgenic studies have indicated that this kinase plays a role in proper skin development and inflammatory responses[18], [19].

The ability of RIP1 and RIP2 to transduce stress signals was shown to rely only partially on their catalytic activities. Just like RIP1, the kinase activity of RIP2 is not required for the NF-κB response or for the activation of JNK and p38 [9], [20], [21]. Recent studies have highlighted the importance of ubiquitination as a key process regulating RIP1 and RIP2′s capacity to activate downstream signaling pathways[22], [23], [24]. Ubiquitination is a post-translational modification involving covalent attachment of ubiquitin, a 76-amino acid polypeptide, to a target protein by a cascade of reactions carried out by the concerted action of the ubiquitin-activating (E1), -conjugating (E2) and -ligating (E3) enzymes[25]. Protein ubiquitination is emerging as a key regulatory mechanism that controls many physiological processes, including protein degradation, cell signaling, DNA damage response, and protein trafficking. This wide range of consequences originates from the ability of ubiquitin to form polymers in which an internal Lys residue of one ubiquitin moiety is attached to the carboxy-terminal residue of another. Primarily, Lys48-linked and Lys63-linked polyubiquitin modifications have been studied, and these linkages are respectively known to be required for proteasomal degradation and signal transmission. Recently, attention has been paid to a new type of ubiquitin chain that plays a role in NF-κB activation – the linear ubiquitin chains, arising from attachment of the C-terminal Gly to the N-terminal Met. So far, LUBAC is the only E3 ubiquitin ligase complex reported to conjugate substrates with linear ubiquitin chains[26], [27].

Inhibitor of apoptosis proteins (IAPs) are phylogenetically conserved proteins characterized by the presence of at least one Baculovirus IAP Repeat (BIR) motif, a zinc-binding structure of approximately 70 amino acid residues that mediates protein-protein interactions[28]. As indicated by their name, the function of these proteins was first believed to be restricted to inhibition of cell death, mostly by direct interference with the proteolytic activities of caspases. However, several studies later proved that IAPs have a much wider spectrum of action, and that XIAP is probably the only member capable of direct caspase inhibition. In addition to their BIR domains, an interesting feature of XIAP, cIAP1 and cIAP2 is the presence of a RING finger domain conferring E3 ubiquitin ligase activity. Cellular IAP proteins can promote Lys48- and Lys63-linked polyubiquitination of RIP1 and RIP2[29], [30], [31], [32]. The addition of Lys63-linked ubiquitin chains to these proteins was reported to create a platform for the recruitment and activation of the TAB-TAK1 complex and of the IκB Kinase (IKK) complex IKKα-IKKβ-NEMO, which drives NF-κB activation and thereby induces gene transcription [22], [23]. However, recent studies have shown that Lys63-linked ubiquitin chains might not be essential for NF-κB activation, suggesting existence of other chain types for the recruitment and activation of the these kinase complexes[35], [36]. Interestingly, cIAP1 has been shown to induce Lys6-, Lys11-, Lys48-, and Lys63-auto-ubiquitination, and a recent study demonstrated the ability of cIAP1 to conjugate RIP1 with Lys11-linked ubiquitin chains[33], [37]. In addition, cIAP1/2-mediated ubiquitination of RIP1 was also reported to prevent RIP1 from integrating and activating death complexes[31], [38].

The molecular mechanisms regulating RIP3 and RIP4 functions remain poorly understood, and whether ubiquitination plays a regulatory function is unknown. Because IAPs were shown to be critical regulators of RIP1 and RIP2, we investigated whether they also participate in the regulation of RIP3- and RIP4-mediated functions. We found that XIAP, cIAP1 and cIAP2 bind to RIP3 and RIP4, and that cIAP1 and cIAP2 function as direct E3-ubiquitin ligases for all four RIP proteins. By performing *in vitro* ubiquitination reactions, we found that cIAP1 not only conjugate RIP proteins with Lys48- and Lys63-linked polyubiquitin chains but also with head-to-tail linear ubiquitin chains. Consistent with these findings, we found that repressing cIAP1/2 levels by the use of the IAP antagonist BV6 attenuated activation of NF-κB that is dependent on RIP1, -2, -3 and -4 (hereafter RIP1–4). Finally, we identified two lysines of RIP4 that play a critical role in cIAP1-mediated ubiquitination and NF-κB activation. Together, our results provide new insights into the regulatory mechanisms controlling the members of the RIP family.

## Materials and Methods

### Plasmids

We are grateful to Prof. J. Tschopp for providing us with the VSV-tagged human RIP1-RIP4 plasmids. The plasmids encoding RIP4 mutants were generated from a pCR3 construct encoding VSV-tagged wild-type human RIP4 by using the QuickChange mutation kit (Stratagene). The primer pairs used to generate the K-R amino acid changes are as follow:

K145R: 5′-ctgcacctggacctcaggccggcgaacatcctgctg-3′ and 5′-cagcaggatgttcgccggcctgaggtccaggtgcag-3′, K158R: 5′-gcccactaccacgtcaggatttctgattttggt-3′ and 5′-accaaaatcagaaatcctgacgtggtagtgggc- 3′, K220-221R: 5′-ggcgtgctcacacagaggaggccgtttgcagatgag-3′ and 5′-ctcatctgcaaacggcctcctctgtgtgagcacgcc-3′

### Pull-down, immunoprecipitation and immunoblotting experiments

Pull-downs: VSV-tagged RIP proteins were produced *in vitro* with a TnT T7 quick-coupled transcription and translation system (Promega, Cat# L-4610) in the presence of ^35^S-methionine (Perkin Elmer, Cat# NEG-709A). GST, GST-XIAP, GST-cIAP1, and GST-cIAP2 fusion proteins were produced in bacteria from pGEX4T2 constructs and purified using glutathione sepharose 4FF beads (Amersham GE Healthcare, Cat# 17-5132-01). Pull-down assays were performed in NP40 buffer (10 mM Tris pH 8.0, 150 mM NaCl, and 1% Nonidet P-40) supplemented with protease and phosphatase inhibitor tablets (Roche, Cat # 11873580001, # 04906837001).

Immunoprecipitation: HEK293T cells[39] were transfected with the appropriate plasmids using a standard calcium phosphate protocol and lysed in NP40 buffer 24 h post-transfection. Coimmunoprecipitations were performed in presence of either anti-FLAG M2 Affinity Gel (beads) (Sigma, Cat# A2220) or protein G sepharose 4FF beads (GE Healthcare, Cat # 17-0618-01) coupled to Myc 9E10 monoclonal antibody (produced in our lab). SDS-polyacrylamide gel electrophoresis and immunoblotting were performed in accordance with standard protocols. For immunoblotting, we used anti-VSV and anti-FLAG antibodies from Sigma (Cat# V 4888, F7425). The anti-cIAP1 antibody was obtained from Dr. J Silke (La Trobe University), and the anti-ubiquitin-HRP antibody was purchased from ENZO Life Sciences – Biomol (Cat# PW0150). Direct immunoprecipitations were performed in RIPA buffer (10 mM Tris [pH 8.0], 150 mM NaCl, 1% Nonidet P-40, 0.1% SDS, and 0.5% deoxycholate).

Lambda-phosphatase treatment: A plasmid encoding Flag-tagged RIP4 was transfected into HEK293T cells[39]. 24h later lysates were made and RIP4 was affinity purified, making use of Flag-M2-beads (Sigma). Afterwards the beads were washed with PBS and incubated in presence or absence of 100U of λ-phosphatase (Biolabs) for 1 hour according to the manufacturer's protocol. Samples were loaded on SDS-PAGE and submitted to western blotting with anti-Flag antibody for detection.

### *In vitro* ubiquitination assays

VSV-tagged RIP proteins were produced *in vitro* with a TnT T7 quick coupled transcription and translation system (Promega, Cat# L-4610) in the presence of ^35^S- methionine (Perkin Elmer, Cat# NEG-709A). GST, GST-XIAP, GST-cIAP1, and GST-cIAP2 fusion proteins were produced in bacteria from pGEX4T2. *In vitro* ubiquitination reactions were carried out at 37°C for 60 min in 75 mM Tris pH 8, 2 mM DTT, 5 mM MgCl_2_, 4 mM ATP, 50 nM E1 (Boston Biochem Cat# E-305), 0.2 mM ubiquitin (WT, K48-only, K63-only, K0, Myc-tagged) (Boston Biochem Cat# U-100H, UM-K480, UM-K630, UM-NOK, U-115), 0.5 µM UbcH5a E2 (Boston Biochem Cat. # E2-616), 0.03 mg/ml E3 and 20% of the Myc-tagged RIP TnT reaction product. After incubation at 37°C for 1h, the reaction products are boiled in presence of Laemmli buffer and run on 8% acrylamide gels. The gels are then fixed (10% acetic acid, 40% Methanol in water), dried and exposed. RIP ubiquitination was revealed by autoradiography and appears as a smear in the figure.

### NF-κB luciferase assays

HEK293T cells[39] were transfected with the indicated expression vectors combined with 100 ng of reporter plasmids for NF-κB-luciferase and pACT-β-galactosidase. Where indicated, 1 µM BV6 was added 8 h after transfection. The cells were stimulated with TNF for 4 h using 10,000 U/ml of hTNF. Twenty-four hours post transfection, the cells were collected, washed in PBS and lysed in Luciferase lysis buffer (25 mM Tris phosphate pH 7.8, 2 mM DTT, 2 mM CDTA, 10% glycerol and 1% Triton-X-100). Substrate buffer was added (658 mM luciferin, 378 mM coenzyme A and 742 mM ATP) and Luciferase activity (Luc) was assayed in a GloMax 96 Microplate Luminometer (Promega). β-Galactosidase (Gal) activity in cell extracts was assayed with chlorophenol red β-D-galactopyranoside substrate (Roche Applied Science, Basel, Switzerland) and the optical density was read at 595 nm in a Benchmark microplate Reader (Bio-Rad Laboratories, Nazareth, Belgium). Luc values were normalized for Gal values to correct for differences in transfection efficiency (plotted as Luc/Gal). The untreated or wild type condition was set as 100. The data represent the average ± S.D. of triplicates.

## Results

### XIAP, cIAP1 and cIAP2 physically interact with RIP3 and RIP4

**Figure 1:**
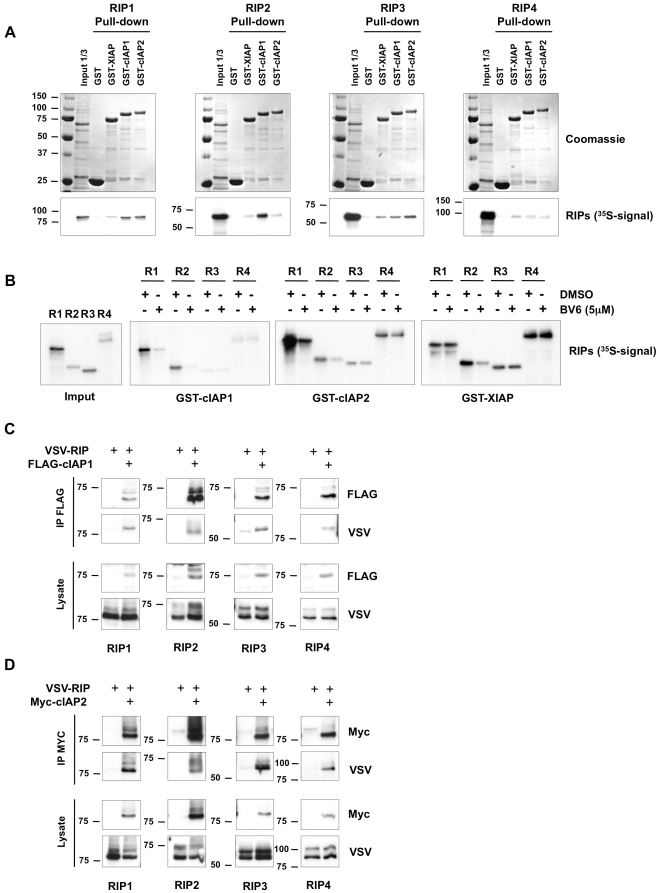
XIAP, cIAP1 and cIAP2 bind RIP1-4 proteins. (A) *In vitro*-transcribed and -translated RIP1, 2, 3 and 4 labeled with ^35^S-methionine (Input) was incubated overnight with bacterially produced GST, GST-XIAP, GST-cIAP1, or GST-cIAP2 bound to Sepharose beads. The beads were washed extensively and run on an acrylamide gel. Binding of RIPs was revealed by autoradiography (pull-down). (B) *In vitro*-transcribed and -translated RIP1, 2, 3 and 4 labeled with ^35^S-methionine (Input) was incubated overnight with bacterially produced GST-XIAP, GST-cIAP1, or GST-cIAP2 bound to Sepharose beads in presence of BV6 (5 µM) or DMSO as a control. The beads were washed extensively and run on an acrylamide gel. Binding of RIPs was revealed by autoradiography. (C) HEK293T cells were transfected with VSV-tagged RIP plasmids in the absence or presence of a FLAG-tagged cIAP1 plasmid. cIAP1 was immunoprecipitated in NP-40 buffer using anti-FLAG antibody and coimmunoprecipitated RIPs were revealed by immunoblotting with anti-VSV antibody. Protein expression in the lysates is shown. (D) HEK293T cells were transfected with VSV-tagged RIP plasmids in the absence or presence of a Myc-tagged cIAP2 plasmid. cIAP2 was immunoprecipitated in NP-40 buffer using anti-Myc antibody and coimmunoprecipitated RIPs were revealed by immunoblotting with anti-VSV antibody. Protein expression in the lysates is shown.

Previous studies have shown that XIAP, cIAP1 and cIAP2 bind to RIP2 by association through the kinase domain[29], [40], which suggests that other members of the RIP family can also bind to these IAPs. To test whether RIP3 and RIP4 directly bind these IAPs, we performed pull-down experiments using bacterially produced GST-XIAP, GST-cIAP1 and GST-cIAP2 fusion proteins and *in vitro*-transcribed and -translated radiolabeled RIP3 and RIP4. Purified GST was used to assess nonspecific binding and RIP1 and RIP2 were used as positive controls. As previously reported, RIP1 interacted with GST-XIAP and displayed even higher affinity for GST-cIAP1 and GST-cIAP2 (Figure 1A). RIP2 bound to GST-cIAP1 and to a lesser extent to GST-XIAP and GST-cIAP2. Interestingly, we found that RIP3 and RIP4 could also directly interact with these three GST-IAPs. Association of cIAP2 and RIP2 was reported to require a two-site interaction involving regions in both the BIR domains and the CARD-RING domain of cIAP2[29]. The SMAC binding site of the BIR2 domain of XIAP was shown to be crucial for RIP2 binding, and the association of XIAP with RIP2 could be inhibited in presence of a SMAC mimetic compound [40]. In order to test whether the use of the SMAC mimetic BV6 could also inhibit binding of the IAPs to the other RIPs, we included BV6 in another set of pull-down experiments[41]. As shown in figure 1B, the presence of BV6 greatly reduced the binding of XIAP to RIP2 but had not impact on the association of XIAP with RIP1, RIP3 or RIP4. The presence of BV6 also greatly inhibited binding of cIAP1 and cIAP2 to RIP1 and RIP2, but had no influence on their association with RIP3 and RIP4. Together, these results indicate that the IAPs use different binding motifs for their association with the different RIPs. In addition, these experiments also provide information on the relative affinity of each IAP for the different RIPs: cIAP1: R1 = R2>R4>R3; cIAP2: R1>R2 = R4>R3; XIAP: R4 = R2>R3>R1.

To address whether cIAP1 and cIAP2 interact with RIP3 and RIP4 also in cells, we performed immunoprecipitation experiments with lysates of HEK293T cells overexpressing the different RIPs in the absence or presence of cIAP1 or cIAP2. As shown in Figures 1C and 1D, all four RIPs co-immunoprecipitated with cIAP1 or cIAP2. The pre-mentioned relative affinity of cIAP2 for RIP1-4 was also confirmed in a cellular context (Figure S1).

### cIAP1 and cIAP2 are direct E3 ubiquitin ligases for RIP1–4

**Figure 2:**
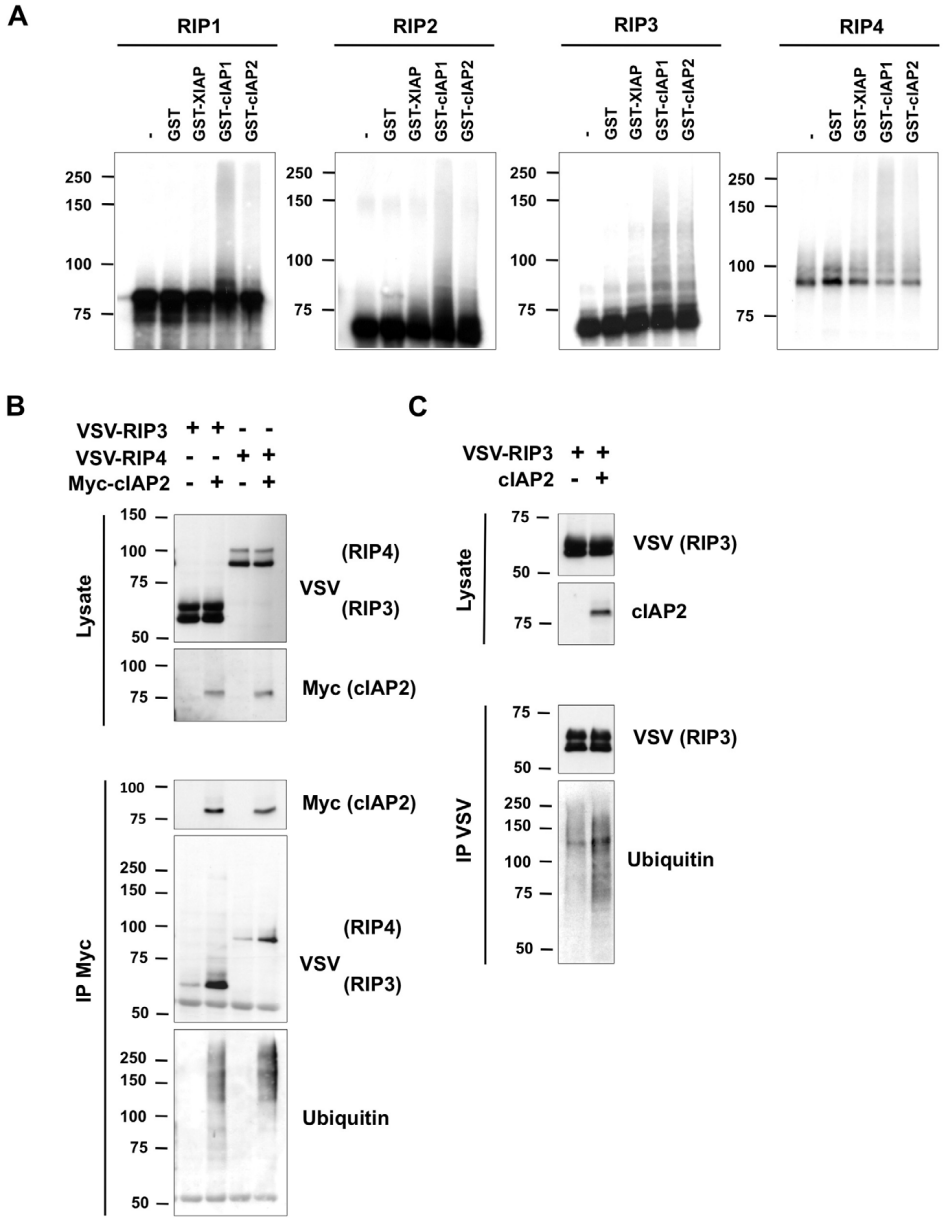
cIAP1 and cIAP2 are direct E3 ubiquitin ligases for RIP1–4 proteins. (A) *In vitro* ubiquitination assays were performed on *in vitro*-transcribed and -translated RIP1–4 proteins labeled with ^35^S-methionine. GST, GST-XIAP, GST-cIAP1, or GST-cIAP2 was used as E3 component, UbcH5a as the E2 component, and using wild-type ubiquitin. RIP ubiquitination was revealed by autoradiography and appears as a smear in the figure. (B) HEK293T cells were transfected with VSV-tagged RIP3 and RIP4 plasmids in the absence or presence of a Myc-tagged cIAP2 plasmid. cIAP2 was immunoprecipitated in NP-40 buffer using anti-Myc antibody and coimmunoprecipitated ubiquitinated RIP3 and RIP4 were revealed by immunoblotting with anti-VSV and anti-ubiquitin antibodies. Protein expression in the lysates is shown. (C) HEK293T cells were transfected with VSV-tagged RIP3 plasmid in the absence or presence of a cIAP2 plasmid. RIP3 was immunoprecipitated in RIPA buffer using anti-VSV antibody and ubiquitinated RIP3 was revealed by immunoblotting with anti-VSV and anti-ubiquitin antibodies. Protein expression in the lysates is shown.

We recently described a crucial role for cIAP1/2 in RIP1- and RIP2-signaling pathways, and showed that cIAP1/2 can act as direct E3 ubiquitin ligases for RIP1[29], [31]. In the current study, we tested whether XIAP, cIAP1 or cIAP2 could also act as direct E3 ligases for RIP2–4 by performing *in vitro* ubiquitination assays. To do so, we incubated ^35^S-methionine-labeled RIPs, transcribed and translated *in vitro*, with bacterially produced GST-XIAP, GST-cIAP1 and GST-cIAP2, and used UbcH5a as E2 component. As shown in Figure 2A, no ubiquitination was observed in absence of E3 enzyme (beads alone (-) or GST alone) and, as previously described, RIP1 was a direct substrate for cIAP1 and cIAP2[30], [31], [32]. Interestingly, we found that addition of GST-cIAP1 also strongly induced ubiquitination of the other three RIPs. The presence of GST-cIAP2 resulted in ubiquitination of RIP2–4, although not as efficiently as GST-cIAP1. On the contrary, incubation with GST-XIAP only led to weak ubiquitination of RIP4.

When ectopically expressed in HEK293T cells, cIAP1/2 induce strong ubiquitination of cIAP2-bound RIP1 and RIP2, detectable by immunoblot using anti-VSV antibody (Figure S1 and data not shown). The use of anti-VSV antibody does not allow detection of ubiquitinated forms of cIAP1/2-bound RIP3 or RIP4, probably due to the lower affinity of cIAP1/2 for these proteins and therefore reaching the antibody detection limits. However, immunoblot using anti-ubiquitin antibody revealed ubiquitin smears with sizes corresponding to RIP3 and RIP4 (Figure 2B). We confirmed cIAP2-mediated RIP3 ubiquitination in cells by direct immunoprecipitation of RIP3 in RIPA buffer (Figure 2C). Together these results demonstrate the role of cIAP1/2 as direct E3 ubiquitin ligases for RIP1–4 *in vitro* and in a cellular context.

### cIAP1 conjugates RIP1–4 with diverse ubiquitin chains, including head-to-tail linear chains

**Figure 3:**
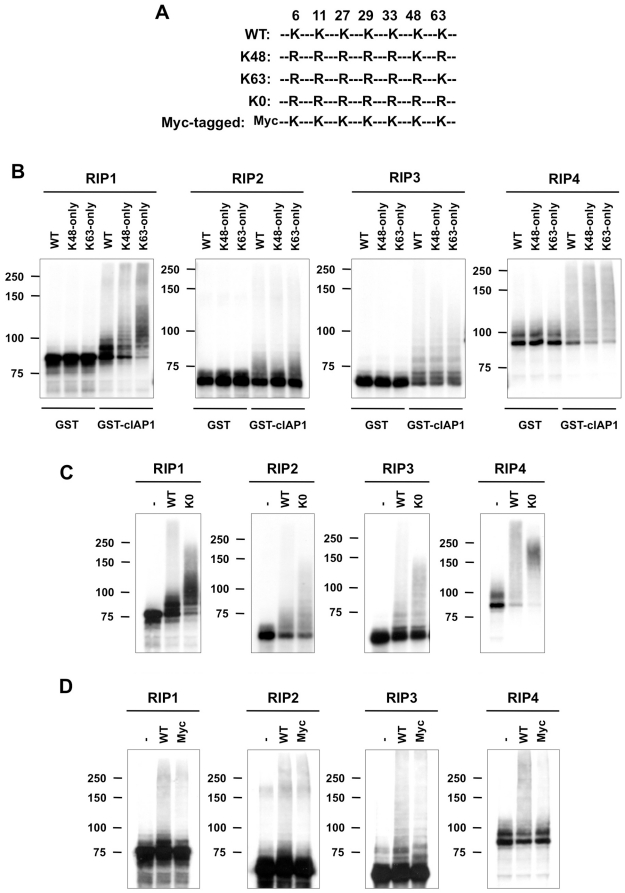
cIAP1 conjugates RIP1–4 proteins with Lys48-, Lys63-, and linear ubiquitin chains. (A) Schematic representation of the different ubiquitin mutants used in the experiments. (B–D) *In vitro* ubiquitination assays were performed on *in vitro*-transcribed and -translated RIP1–4 proteins labeled with ^35^S-methionine. GST or GST-cIAP1 was used as E3 component and UbcH5a as the E2 component. The reaction was carried out in presence of (B) wild-type (WT) ubiquitin, K48-only ubiquitin and K63-only ubiquitin; (C) no ubiquitin, wild-type (WT) ubiquitin and lysine free ubiquitin (KO); (D) no ubiquitin, wild-type (WT) ubiquitin and Myc-tagged ubiquitin. Ubiquitination of the RIPs was revealed by autoradiography.

To identify the type of ubiquitin chains conjugated by cIAP1/2 to the RIPs, we next performed series of *in vitro* ubiquitination reactions using ubiquitin variants in which all Lys residues, except Lys48 or Lys63, were mutated (Figure 3A–B). Because cIAP1 ubiquitinates RIP1–4 more efficiently than cIAP2 *in vitro*, we decided to concentrate our work on cIAP1. We found that cIAP1 could induce RIP-ubiquitination irrespectively of the type of ubiquitin variant used (Figure 3B). This implies that cIAP1 mediates ligation of both Lys48- and Lys63-ubiquitin linkages on RIP1-4, or, alternatively, that it mediates linear ubiquitination. To test for the presence of head-to-tail linkages, we performed ubiquitination assays using a Lys-free ubiquitin variant (KO). Interestingly, we found that cIAP1-mediated ubiquitination of the RIPs was not inhibited when using this mutant, which suggests the presence of linear ubiquitin chains (Figure 3C). Because multi mono-ubiquitination is another potential explanation for our observations, we repeated the experiment using Myc-tagged ubiquitin. This variant does not affect mono-ubiquitination but inhibits linear chain formation. Figure 3D shows that the use of Myc-tagged ubiquitin did not inhibit, but repressed cIAP1-mediated ubiquitination of RIP1 and RIP4. On the other hand, it only had a minor effect on RIP2 and RIP3 ubiquitination. Taken together, our data demonstrate that cIAP1 and cIAP2 are direct E3 ubiquitin ligases for all four RIPs and indicate that cIAP1 conjugates RIP1–4 with diverse types of ubiquitin chains, including linear chains.

### cIAP1/2 regulate NF-κB activation mediated by RIP1–4

**Figure 4:**
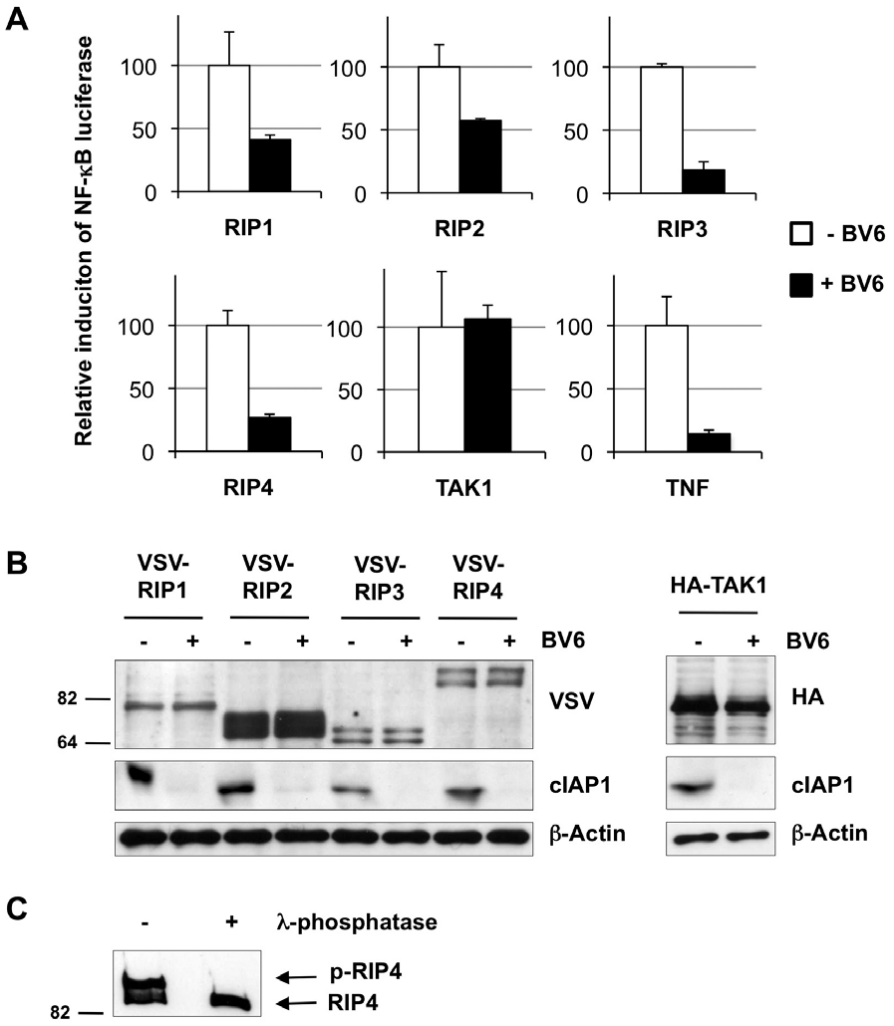
Requirement of cIAP1/2 for RIP1–4-mediated NF-κB activation. (A) NF-κB luciferase assays were performed on lysates from HEK293T cells transfected with a plasmid encoding the indicated RIP kinase or TAK1. Cells were left untreated or were treated with 1 µM of BV6. Stimulation with hTNF was used as a positive control. (B) Western blot showing expression of the tagged-RIP kinases, cIAP1 and TAK1 in the lysates. (C) Phosphorylation of RIP4 was revealed by λ-phosphatase treatment of immunopurified Flag-RIP4 overexpressed in HEK293T cells. Samples were analyzed by western blot using anti-Flag antibody.

The RIP kinases are involved in several cellular signaling pathways regulating differentiation, inflammatory responses, and cell death or survival[42]. A commonality of RIP1–4 is their role in activating the canonical NF-κB pathway when ectopically expressed. Lys63-ubiquitination has been reported to be crucial for this mediatory function of RIP1 and RIP2 because it promotes assembly of a pro-survival signaling platform that facilitates TAK1-dependent phosphorylation of the IKKα/IKKβ complex[22], [23]. cIAP1/2 are positive regulators of TNFR1- and NOD1/2-induced canonical NF-κB pathway, and are required for Lys63-ubiquitination of RIP1 and RIP2 [29], [30], [31], [43]. To test whether RIP3 and RIP4 have to be ubiquitinated by cIAP1/2 in order to mediate NF-κB activation, we compared RIP-mediated NF-κB luciferase reporter activity when ectopically expressed in HEK293T cells in the presence or absence of the IAP inhibitor BV6, a treatment that induces rapid auto-ubiquitination and degradation of endogenous cIAP1/2[41]. As shown in Figure 4A, BV6 treatment greatly impaired TNF and RIP1–RIP4-induced NF-κB activation but had no impact on TAK1-mediated NF-κB induction (Figure 4A). Those results, which indicate that cIAP1/2 act upstream of TAK1, are consistent with a role for cIAP1/2 as E3 ligases regulating RIP1–4-mediated activation of NF-κB. Ectopic expression of the RIPs has been reported to induce their auto-phosphorylation and we confirmed phosphorylation of RIP4 by λ-phosphatase treatment (Figure 4C). It is important to note that BV6 treatment did not affect RIP or TAK1 protein expression levels nor modified the phospho-status of the overexpressed RIPs (Figure 4B). Together, our results demonstrate that depleting cIAP1/2 inhibits RIP1-4 mediated NF-κB activation without affecting RIP auto-phosphorylation.

**Figure 5:**
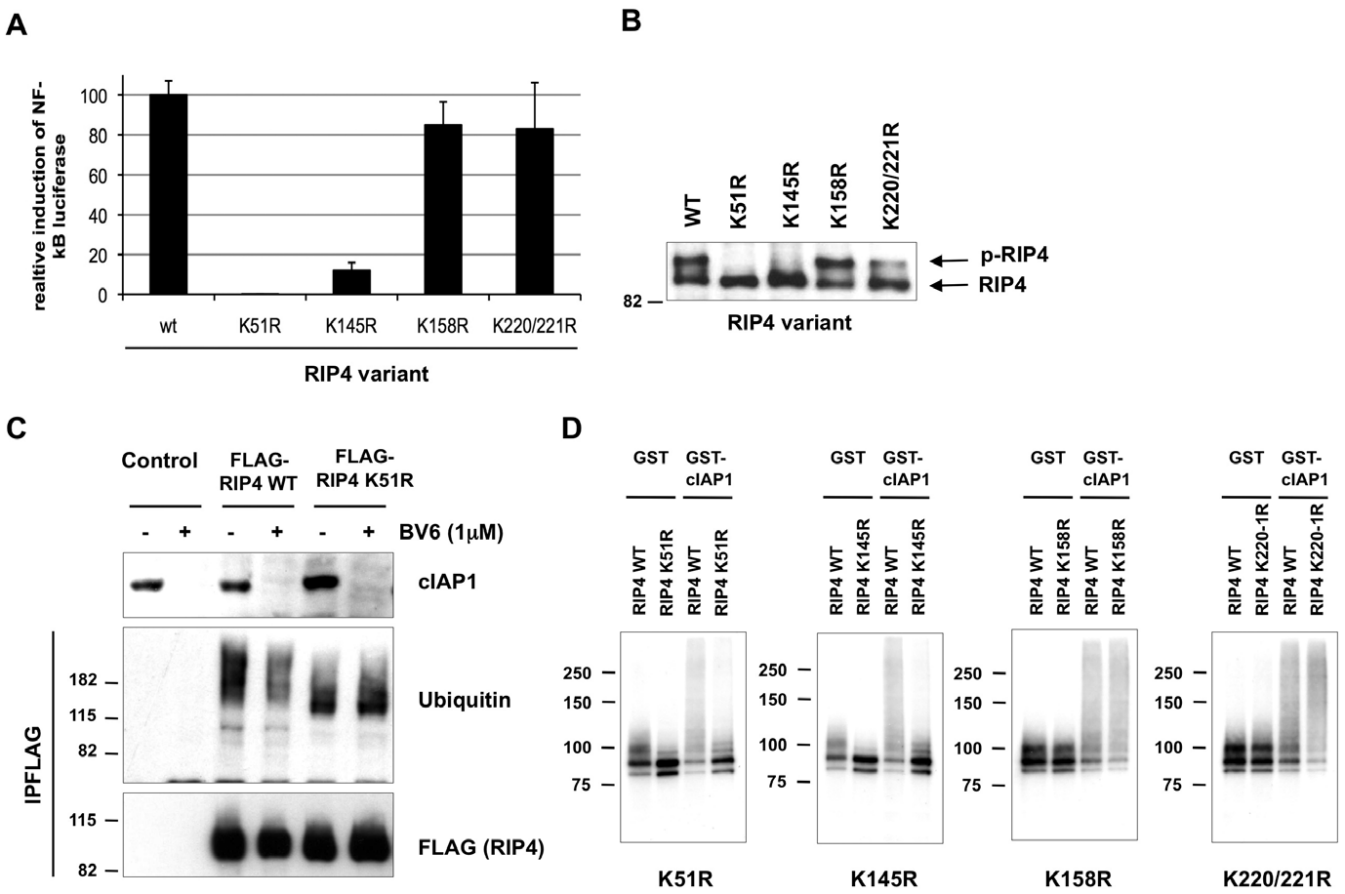
Lysine residues K51 and K145 of RIP4 are critical for cIAP1-mediated ubiquitination and NF-κB activation. (A) Flag-tagged wild type RIP4, K51R, K145R, K158R and K220/221R mutants were ectopically expressed in HEK293T cells together with a NF-κB luciferase reporter. 24h after transfection, lysates were prepared and tested for luciferase activity. Relative luciferase activity is depicted after correction for transfection efficiency, ± S.D. (B) Lysates described in (B) were used for western blotting analysis with an anti-Flag antibody in order to detect expression of the different Flag-tagged RIP4 variants. (C) HEK293T cells were transfected with FLAG-tagged wild-type RIP4, the K51R mutant or with empty vector. The medium was replaced 8h post-transfection with medium containing, or not, BV6 (1 µM). The RIP4 proteins were immunoprecipitated 18h later in RIPA buffer using anti-FLAG antibody and ubiquitinated RIP4 proteins were revealed by immunoblotting with anti-FLAG and anti-ubiquitin antibodies. (D) *In vitro* ubiquitination assays were performed on *in vitro*-transcribed and -translated wild-type RIP4, K51R, K145R, K158R and K220/221R mutants labeled with ^35^S-methionine. GST or GST-cIAP1 was used as E3 component and UbcH5a as E2 component. The reaction was carried out in presence of wild-type ubiquitin. RIP4 ubiquitination was revealed by autoradiography.

Previous studies identified Lys377 in the intermediate domain (ID) of RIP1 and Lys209 in the kinase domain (KD) of RIP2 as important residues acting as acceptor sites for Lys63-ubiquitination and NF-κB activation[22], [23]. Other studies identified the C-terminal region of RIP3 (excluding the KD) and the KD of RIP4 as sufficient for NF-κB activation[15], [16], [17], indicating that RIP proteins do not seem to use conserved residues in order to activate NF-κB. These observations prompted us to look for alternative ubiquitin acceptor sites on RIP3 and RIP4. Because RIP3 is a weak inducer of NF-κB, we decided to limit our study to RIP4 and generated a series of full-length RIP4 variants containing a Lys residue mutation within the KD. We mutated Lys51 (the ATP-binding pocket), Lys145, Lys158 (upstream of the RIP4 T-loop) or Lys220/221 to arginines and then tested the ability of these mutants, when expressed ectopically, to activate NF-κB. As shown in Figure 5A, expression of K158R and K220/221R mutants induced NF-κB activation similarly to full-length wild-type RIP4. On the contrary, mutation of Lys51 (K51R) or Lys145 (K145R) strongly repressed NF-κB activation. Figure 5B shows that all mutants were expressed at similar levels and that overexpression of K51R and K145R did not induce phosphorylation of RIP4. To correlate these defects of NF-κB activation with defects of ubiquitination, we ectopically expressed wild-type RIP4 and the K51R mutant in HEK293T cells and compared their ubiquitination status by immunoblot after immunoprecipitation. Surprisingly, we found that both RIP4 proteins were highly ubiquitinated, although with different ubiquitination patterns (Figure 5C). However, BV6 treatment repressed ubiquitination of the wild-type RIP4 but not of the K51R mutant. Remarkably, *in vitro* ubiquitination assays revealed that cIAP1-mediated ubiquitination of RIP4 was greatly affected when using K51R or K145R but not when using K158R or K220/221R (Figure 5D). Together, our results show that cIAP1/2 regulates RIP1–4-mediated activation of NF-κB and identify Lys51 and Lys145 of RIP4 as important residues for cIAP1-mediated ubiquitination and NF-κB activation.

## Discussion

Among the eight IAP members encoded by the human genome, five contain a carboxy-terminal RING domain that provides them with E3 ubiquitin ligase activity, and two (cIAP1 and cIAP2) were shown to regulate RIP1 and RIP2 functions by conjugating them with ubiquitin chains[28], [29], [30], [31]. XIAP, another RING domain containing IAP, has also been shown to regulate RIP2-mediated functions, but whether this requires its ligase activity is unknown[40]. In this study, we report that in addition to RIP1 and RIP2, also RIP3 and RIP4 directly interact with XIAP, cIAP1 and cIAP2. When comparing the ability of these IAPs to directly conjugate RIP1–RIP4 with ubiquitin chains, we found that cIAP1 was the most effective E3 and was capable of ubiquitinating all four RIPs in the presence of the E2 component UbcH5a. On the contrary, XIAP was only capable of inducing weak ubiquitination of RIP4. However, because other E2 components or additional adaptor proteins might be required for XIAP to function, we cannot exclude its potential role as E3 for the RIPs in physiological settings. The consequence of ubiquitination depends on the type of ubiquitin chains added to the substrate: Lys48-ubiquitin chains direct proteins towards proteasomal degradation whereas Lys63- and linear chains act as docking sites for the activation of signaling pathways[25]. Using ubiquitin mutants, we were surprised to find that cIAP1 conjugates the RIPs not only with Lys48- and Lys63-ubiquitin chains but also with linear chains. The addition of Lys63-ubiquitin chains to RIP1 and RIP2 was reported to create a platform for the recruitment of the TAB-TAK1 and IKKα-IKKβ-NEMO complexes[22], [23]. The close proximity created on the ubiquitin chains between TAK1 and IKKβ is believed to be sufficient for TAK1 to activate IKKβ by phosphorylation and therefore activate the NF-κB pathways, implying that TABs and NEMO ubiquitin binding domains (UBD) are specific for Lys63 chains. However, recent studies indicated that Lys63-ubiquitin chains might not be essential for TNF-induced NF-κB activation, and that NEMO possesses high affinity for other ubiquitin chains, including Lys11- and linear chains[33], [35], [36], [44]. Consistent with this, mass spectrometry analysis revealed that RIP1 is conjugated with Lys11-, Lys48-, Lys63- and linear ubiquitin chains in the TNFR signaling complex[33], [34]. Interestingly, Dynek et al. reported cIAP1-mediated Lys11-ubiquitination of RIP1[33], and we now provide evidence for cIAP1-mediated linear ubiquitination of RIP1. LUBAC is the only E3 complex identified, so far, capable of linear ubiquitination[26]. Although LUBAC strongly ubiquitinates NEMO *in vitro*, its effect on RIP1 are rather minor[34], suggesting the existence of other linear-ubiquitinating enzymes. In our study, we show that cIAP1 strongly ubiquitinates RIP1 when using the K0 ubiquitin mutant *in vitro*, suggesting linear ubiquitin chain conjugation. The reduced ubiquitination observed when using the Myc-tagged form of ubiquitin supports this idea. However, mass spectrometry analysis would be required to clearly identify those chains as being linear. The finding that cIAP1 conjugates RIPs with diverse ubiquitin chains provides new interpretations of how receptor-signaling complexes might be assembled. We found that, unlike other RIPs, cIAP1 mediates mostly Lys63-ubiquitination of RIP2.

Activation of the NF-κB pathway is a property of all RIP family members, and we used it as a functional read-out to test the role of cIAP1/2 in the regulation of RIPs functions. Depletion of cIAP1/2 by BV6 treatment greatly affected RIP1–4-dependent NF-κB activation, which confirms a role for cIAP1/2 in regulating RIPs functions. We and others previously reported that cIAP-mediated ubiquitination of RIP1 prevents RIP1 from integrating and activating death complexes – either apoptotic or necrotic[24], [30], [31], [38], [43], [45]. The physiological relevance of the regulation of RIP3 functions by cIAP1 and cIAP2 might therefore go beyond NF-κB activation. Indeed, several recent publications have highlighted the crucial role of RIP3 in the necrotic cell death pathways, and we recently reported that cIAP1/2 depletion by BV6 treatment facilitates RIP1/3-necrotic complex formation and cell death[11], [12], [13], [38], [45]. Because RIP1 is implicated in many of the RIP3-dependent necrotic pathways[46], it will be important in the future to study the role of cIAP1 in the regulation of RIP1-independent but RIP3-mediated necrosis. A potential model system for this is the recently reported role of RIP3 during murine cytomegalovirus infection[47].

RIP4 was initially identified as a PKC-interacting kinase[48], [49]. The effect of genetic ablation of RIP4 in the mouse points to a role in skin development[18], whereas skin-specific transgenic mice show increased inflammatory responses[19]. Because no receptor acting upstream of RIP4 has been identified yet, signaling studies on RIP4 have been limited to overexpression paradigms. When ectopically expressed, RIP4 leads to the activation of NF-κB and JNK signaling pathways, which was reported to dependent on its kinase activity[16], [17]. In this study, we found that cIAP1 acts as a direct E3 ubiquitin ligase for RIP4 and that mutation of lysine residues K51 and K145 abrogates cIAP1-mediated ubiquitination of RIP4 and NF-κB activation. We found that both wild-type RIP4 and the K51R mutant are highly ubiquitinated when ectopically expressing in HEK293T cells, although with different ubiquitination patterns (Figure 5C), and that cIAP1/2 depletion only repressed wild-type RIP4 ubiquitination. These results indicate that RIP4 is ubiquitinated by several E3 ubiquitin ligases when ectopically expressed in cells, and that only chains added to certain Lys residues play a role in NF-κB activation. cIAP1 and cIAP2 were originally identified as binding partners for the TNF-associated factors 1 and 2 (TRAF1 and TRAF2)[50], and several studies have suggested that cIAP1, cIAP2, and TRAF2 functionally interact[51], [52], [53], [54], [55]. Interestingly, RIP4 binds several members of the TRAF protein family, and dominant negative TRAF1, TRAF3 and TRAF6 inhibit RIP4-induced NF-κB activation[17]. In addition, a link between PKC kinases and TRAF proteins in IKK complex activation was recently reported[56]. This observation suggests that all these proteins might act together to mediate RIP4-dependent IKK complex activation. However, defects in skin differentiation have not been reported in cIAP1 or cIAP2-deficient mice, which might be explained by redundancy between cIAP1/2[57]. Simultaneous deletion of cIAP1 and cIAP2 in keratinocytes *in vivo* should provide insights in their physiological roles in regulating RIP4 function.

Consistent with previous studies, we show that the K51R mutant, which replaces the essential lysine residue in the conserved ATP-binding site resulting in loss of RIP4 kinase activity, is defective for NF-κB activation[16], [17]. We found that the K145R mutation, which also inhibits RIP4 auto-phosphorylation (Figure 4B–C), similarly inhibited NF-κB activation. Remarkably, we observed that both mutants are also defective in cIAP1-mediated ubiquitination, therefore raising the question about the relative contribution of ubiquitination and RIP4 kinase activity in the activation of the NF-κB response. We found that depletion of cIAP1/2 by BV6 treatment greatly repressed RIP4 ubiquitination and RIP4-mediated NF-κB activation without affecting RIP4 auto-phosphorylation (Figure 4B–5C). Together, these results suggest that RIP4 auto-phosphorylation might be an early signal required for cIAP1 to mediate RIP4-dependent NF-κB activation. In accordance with this view, it is therefore possible that Lys51 and Lys145 are not acceptor sites for ubiquitination. However, both residues are conserved between human RIP1-4 proteins, and Lys140 of RIP1 (homologous to Lys145 of RIP4) has previously been identified as ubiquitin acceptor site by mass spectrometry[58], supporting Lys145 of RIP4 as a potential ubiquitin acceptor site. Contrary to RIP4, those two residues are most probably not important for RIP1 and RIP3-mediated NF-κB activation since this mediatory function was reported to depend on the ID of RIP1 and on the c-terminal portion of RIP3[23], [59]. In addition, RIP3 K50D mutant (homologous to RIP4 K51R) does not show defect in NF-κB activation[15]. On the other hand, it might be interesting in future work to test the effect of mutating the homologue residues in RIP2 since the KD of RIP2 is essential for NF-κB activation[22].

In conclusion, this work demonstrates the role of cIAP1/2 as general regulators of RIP-mediated functions. The fact that cIAP1 conjugates RIP proteins with L48-, Lys63- and linear ubiquitin chains provides new interpretations of how signaling complexes might be recruited to the RIPs by ubiquitination. In addition, those findings suggest new physiological roles for the cIAP1/2 in RIP3 and RIP4 signaling pathways.

## Supporting Information

Figure S1 **HEK293T cells were transfected with VSV-tagged RIP plasmids in the absence or presence of a Myc-tagged cIAP2 plasmid.** cIAP2 was immunoprecipitated in NP-40 buffer using anti-Myc antibody and coimmunoprecipitated RIPs were revealed by immunoblotting with anti-VSV antibody. Protein expression in the lysates is shown.
(TIF)
