# Supplementary figures and images for: cIAP1/2 Are Direct E3 Ligases Conjugating Diverse Types of Ubiquitin Chains to Receptor Interacting Proteins Kinases 1 to 4 (RIP1–4)
Source: PLoS One. 2011 Sep 12;6(9):e22356. doi: 10.1371/journal.pone.0022356 (PMC3171409; doi:10.1371/journal.pone.0022356)

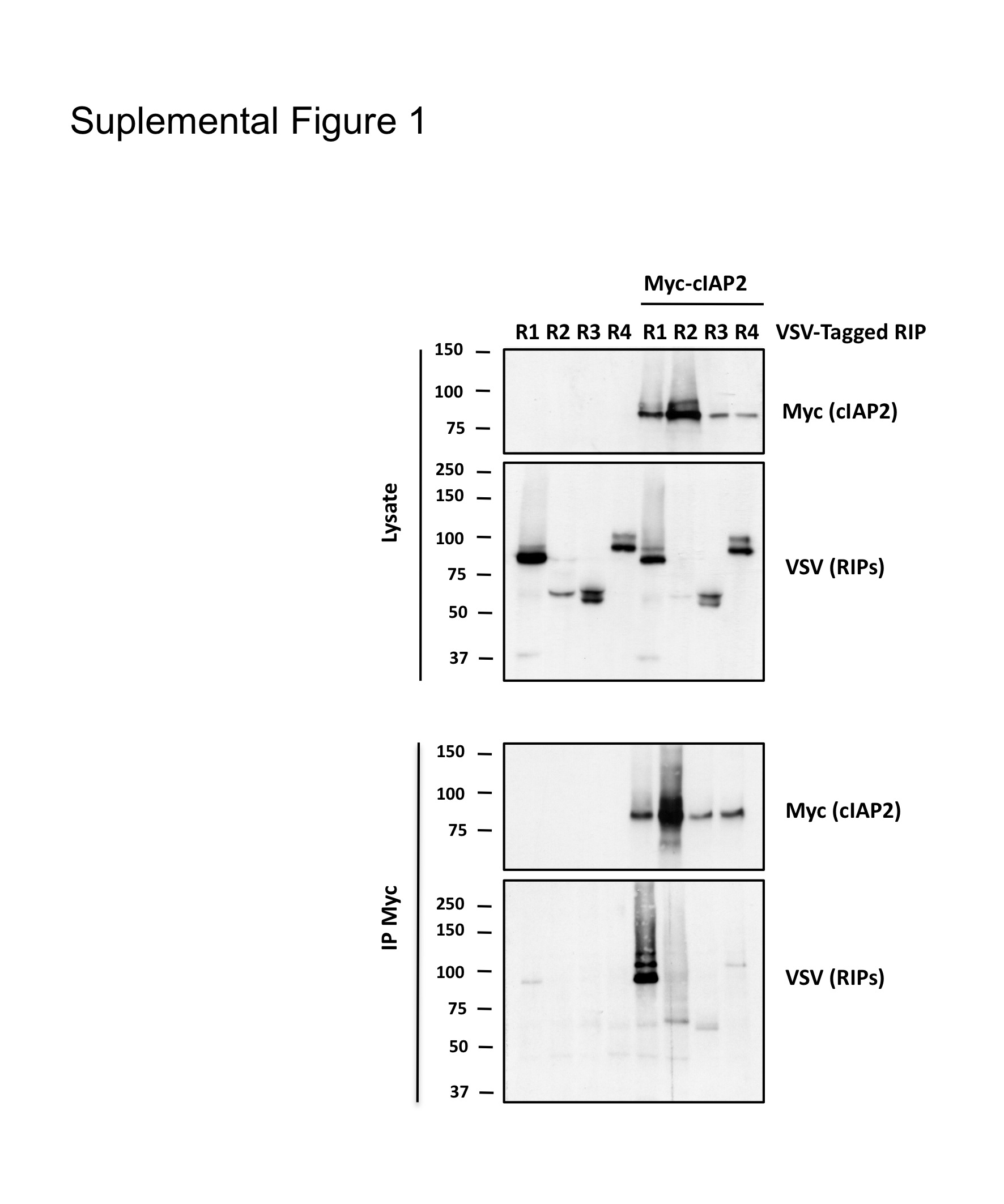

Supplement: Figure S1 — HEK293T cells were transfected with VSV-tagged RIP plasmids in the absence or presence of a Myc-tagged cIAP2 plasmid. cIAP2 was immunoprecipitated in NP-40 buffer using anti-Myc antibody and coimmunoprecipitated RIPs were revealed by immunoblotting with anti-VSV antibody. Protein expression in the lysates is shown. (TIF) [file pone.0022356.s001.tif]
